# Gender differences among ischemic heart disease patients enrolled in a cardiac rehabilitation program

**DOI:** 10.1186/s43044-020-00052-6

**Published:** 2020-03-30

**Authors:** Ahmed Mohamed El Missiri, Hany Mohamed Awadalla, Mosadaq Mustafa Almoudi

**Affiliations:** Cardiology Department, Faculty of Medicine, Ain Shams University, Abbassia square, Abbasia, Cairo, 11566 Egypt

**Keywords:** Gender, Ischemic heart disease, Cardiac rehabilitation, Cardiac rehabilitation program

## Abstract

**Background:**

Cardiac rehabilitation programs reduce cardiovascular events and mortality in ischemic heart disease patients. The aim of this study was to assess gender differences among ischemic heart disease patients enrolled in a cardiac rehabilitation program regarding adherence to the program, as well as, changes in clinical, laboratory, and echocardiographic parameters.

**Results:**

A prospective study that included 30 men and 30 women with stable ischemic heart disease who had been totally revascularized by percutaneous coronary intervention. Patients were enrolled in a 12-week cardiac rehabilitation program. Assessment of demographics, anthropometric measurements, risk factors, and functional capacity was performed. Lipid profile, glycated hemoglobin, and left ventricular ejection fraction were assessed. Assessments were performed at baseline and after completion of the program.

Time to enrollment in the program was prolonged for women 39.17 ± 40.49 vs. 19.77 ± 10.26 days (*p* = 0.014). At baseline, more women were diabetic (*p* = 0.004), hypertensive (*p* = 0.02), had a larger waist circumference (*p* = 0.022), a higher BMI (*p* = 0.011), and higher HbA1c (*p* = 0.033). More men were active smokers (*p* < 0.001). After completion of the program, it was found that men attended 19.1 ± 4.77 (79.6%) sessions compared to 15.7 ± 5.72 (65.4%) sessions for women (*p* = 0.015). Women had more reduction in diastolic BP − 10.93 ± 8.94 vs. − 5.47 ± 12.57 mmHg (*p* = 0.058). The magnitude of reduction in resting heart rate was significant in men (*p* = 0.018) but not in women (*p* = 0.376). The magnitude of reduction in serum total cholesterol and triglycerides was more in men (*p* = 0.018 and *p* = 0.014). Women showed more reduction in HbA1c (*p* = 0.052).

**Conclusion:**

Men are more adherent to cardiac rehabilitation programs. Recruitment of women is significantly delayed. Women have a higher cardiovascular risk burden in the form of prevalence of diabetes, hypertension, and obesity. Completion of a cardiac rehabilitation program causes a reduction in BMI, waist circumference, blood pressure measurements, total cholesterol, triglycerides, LDL-C, HDL-C, HbA1c, and LVEDD with an increase in LVEF in both genders. Men show more reduction in resting HR, total cholesterol, and triglyceride levels while women show more reduction in diastolic BP and HBA1c.

## Background

Cardiovascular disease is the leading cause of death among men and women in most developed countries. Historically, it was thought that cardiovascular disease mainly affects men. This is a myth. Cardiovascular disease is the leading cause of death for women in the United States and represented 54% in 2009 [1–3].

Women and men share the same traditional risk factors for ischemic heart disease. However, women have other non-traditional risk factors such as those caused by premature menopause, pre-eclampsia, depression, social and vocational disparity, and higher prevalence of autoimmune diseases. Additionally, ischemic heart disease symptoms in women are usually atypical and different from men which leads to delayed presentation, delayed diagnosis or misdiagnosis thus putting women at an increased risk. Women usually present more complications and worse functional outcomes compared to men [4–6].

Women are under-represented in clinical research performed in all aspects of cardiovascular disease. The representation of women in clinical trials is still low at around 31%. Action has been taken to try to fix this. However, since women present with cardiovascular disease nearly 10 years later than men, the incidence of cardiovascular disease shows a decline in men and a rise in women mainly caused by a decrease in ischemic heart disease in younger men and its increase in older women [7–10].

Cardiac rehabilitation programs are tailored exercise, educational, and risk factor modification programs. They have been shown to be associated with a significant reduction in cardiovascular events and mortality in ischemic heart disease patients, as well as, an improvement in functional capacity, and psychological and social wellbeing [11, 12].

The aim of this study was to assess gender differences among ischemic heart disease patients enrolled in a cardiac rehabilitation program regarding adherence to the program, as well as, changes in clinical, laboratory, and echocardiographic parameters.

## Methods

This was a prospective study conducted in the period from January 2017 to January 2018. Approval of the institutional ethical committee was obtained, and informed consent was provided by all participants.

This study included 60 consecutive adult patients (30 men and 30 women) with ischemic heart disease who had been totally revascularized by percutaneous coronary intervention (PCI) in the 3 months prior to their enrollment in the study. All patients were angina-free and on standard anti-ischemic medication at the maximum tolerated doses (it was mandatory that the prescribed doses of lipid-lowering medications remain unchanged during the study period). We enrolled all patients in the 12-week cardiac rehabilitation program provided at our institution.

Subjects were excluded from the study if any of the following conditions were met: age less than 18 years old; ongoing exertional or rest angina; recent (less than 1 month) myocardial infarction or acute coronary syndrome; New York Heart Association (NYHA) functional class 3 or 4 [13]; left ventricular (LV) ejection fraction (EF) less than 40%; decompensated heart failure; moderate or severe valvular stenosis or more than moderate valvular regurgitation; hypertrophic obstructive cardiomyopathy; symptomatic hypotension; acute illness; second- or third-degree atrioventricular block; previously implanted pacemaker or implantable cardioverter defibrillator; physical or mental disability that impedes proper exercise training; prior participation in a cardiac rehabilitation program; or the presence of other debilitating conditions such as chronic renal failure, liver cirrhosis, or cerebrovascular stroke.

### Baseline clinical assessment

Thorough history was taken, and detailed clinical examination was performed to obtain each patient’s baseline demographic information, risk factors for ischemic heart disease, as well as, occupational and marital status. Educational level was identified and classified as either being illiterate or having school-level or university-level education. NYHA functional class was identified for all patients. Height in meters and weight in kilograms were recorded. Body mass index (BMI) was calculated according to the formula: BMI = weight in kilograms/square of height in meters (kg/m^2^) [14]. Waist circumference in centimeters was measured using a tape measure midway between the level of the lower costal margin and the level of the anterior superior iliac spines with the patient standing facing forwards in a relaxed position at the end of expiration.

### Laboratory tests

A venous sample was obtained from all patients at baseline to assess 12-h fasting lipid profile to measure total cholesterol, serum triglycerides, and high-density lipoprotein cholesterol (HDL-C) which were assessed using enzymatic methods. While low-density lipoprotein cholesterol (LDL-C) was calculated using the Friedewald formula, where LDL-C = [total cholesterol] − [HDL-C] − (1/5 triglycerides) [15]. Glycated hemoglobin (HbA1c) level was also assessed.

### Standard trans-thoracic echocardiography

Standard trans-thoracic echocardiography was performed for all patients while lying in the left lateral decubitus position using a Vivid S5 machine (GE Vingmed, Netherlands) and an M4S matrix array probe with a frequency range 1.7–3.5 MHz by an echocardiographer accredited from the European Association of Cardiovascular Imaging.

Using m-mode echocardiography from the parasternal short-axis view at the level of papillary muscles we measured LV end-diastolic diameter (LVEDD) and end-systolic diameter (LVESD). LVEF was measured using the biplane Simpson’s method of discs by 2D echocardiography from the apical 4- and 2-chamber views [16, 17].

### Cardiac rehabilitation program

The cardiac rehabilitation program provided at our institution is a 12-week (24 sessions) multi-disciplinary program that includes cardiologists, physiotherapists, psychologists, diabetologists, and trained nurses.

Patients attend sessions for risk factor education and modification; sessions on the importance of medication adherence and their potential adverse events; nutritional counseling classes; smoking cessation classes; group and individual stress management classes; and individual sexual counseling sessions.

The cornerstone of this program is progressive prescribed exercise training. Exercise sessions are held twice weekly. Each patient’s symptoms are evaluated at each visit and the medications’ list is revised. Exercise sessions are similar for all and consist of graded treadmill exercise. A session may last up to 60 min towards the end of the program including warm-up, aerobic training, and cool down periods.

A symptom-limited exercise testing was performed at baseline using the modified Bruce protocol to identify the patient’s maximal heart rate after which the target heart rate was calculated using the Karvonen formula with an exercise intensity of 60% and 75% of heart rate reserve.

Karvonen formula for determination of training heart rate is as follows:

Target heart rate = (heart rate reserve × training percentage required) + resting heart rate

Where heart rate reserve = maximal heart rate − resting heart rate [18].

We assessed the “time to enrollment” for all patients as the time that passed from informing the patient about the program (which was on the day after complete revascularization) to actually joining the program.

### Assessments following completion of the cardiac rehabilitation program

Within a week after completion of the program, patients were interviewed to re-assess smoking status, NYHA functional class, blood pressure, resting HR, body weight, BMI, fasting lipid profile, HbA1c, and the same echocardiographic measurements (LVEDD, LVESD, and LVEF).

Adherence to the cardiac rehabilitation program was assessed using the number (and percentage) of sessions actually attended from the total 24 sessions by each patient.

### Statistical analysis

Data were collected, revised, coded, and entered into the IBM statistical package for social science (SPSS) version 24. Data were examined for normality. Categorical data were presented as number and percentages, while continuous data were presented as mean and standard deviation. Categorical data were compared using chi-square test or Fisher’s exact test when chi-square was not applicable. Continuous data were compared using paired sample *t* test for normally distributed data and independent sample *t* test otherwise. Level of significance (*p* value) was set at *p* < 0.05.

## Results

### Baseline demographic and clinical data

On comparing men and women at baseline, we found that the time to enrollment in the cardiac rehabilitation program was prolonged for women 39.17 ± 40.49 vs. 19.77 ± 10.26 days (*p* = 0.014); a larger proportion of women were diabetic (*p* = 0.004) and hypertensive (*p* = 0.02); women had a larger waist circumference 108.43 ± 8.31 vs. 102.07 ± 12.3 cm (*p* = 0.022) and higher BMI 32.67 ± 5.3 vs. 29.47 ± 4.07 kg/m^2^ (*p* =0.011). On the other hand, current smoking was more common amongst men (*p* < 0.001); it was more common for men to be employed (80%) and for women to be unemployed (56.77%), *p* = 0.03.

There was no difference between men and women at baseline regarding age, presence of dyslipidemia, distribution of NYHA functional class, blood pressure measurements, resting heart rate, marital status, and level of education (Table 1).

**Table 1:** Baseline clinical findings and measurements

Variable	Men (*n* = 30)	Women (*n* = 30)	*p* value
Age, years	53.57 ± 7.77	51.67 ± 6.62	0.312
Time to enrolment, days	19.77 ± 10.26	39.17 ± 40.49	0.014
Current smoker, *n* (%)	29 (96.67%)	12 (40%)	< 0.001
Hypertension, *n* (%)	11 (36.67%)	20 (66.67%)	0.02
Diabetes, *n* (%)	7 (23.33%)	18 (60%)	0.004
Dyslipidemia, *n* (%)	4 (13.33%)	9 (30%)	0.117
BMI, kg/m^2^	29.47 ± 4.07	32.67 ± 5.3	0.011
Waist circumference, cm	102.07 ± 12.3	108.43 ± 8.31	0.022
Marital status—married, *n* (%)	28 (93.33%)	26 (86.67%)	0.704
Level of educational, *n* (%) Illiterate School level education University level education	5 (16.67%) 15 (50%) 10 (33.33%)	6 (20%) 12 (40%) 12 (40%)	0.351
Employed, *n* (%)	24 (80%)	13 (43.33%)	0.003
NYHA functional class, *n* (%) Class 1 Class 2	19 (63.33%) 11 (36.67%)	15 (50%) 15 (50%)	0.435
Systolic BP, mmHg	121.17 ± 14.6	128.83 ± 7	0.066
Diastolic BP, mmHg	76.17 ± 10.14	80.3 ± 7.61	0.079
Resting heart rate, bpm	73.1 ± 9.69	75.43 ± 10.91	0.385

Continuous variables are expressed as mean and standard deviation whereas categorical variables are expressed as number (percentage). *BMI* means body mass index, *NYHA* means New York heart association, *BP* means blood pressure

### Baseline laboratory and echocardiographic measurements

At baseline, HbA1c was significantly higher in women 7.43 ± 2.63 vs. 6.14 ± 1.63% (*p* = 0.033). There was no difference between men and women in serum total cholesterol, triglycerides, LDL-C, and HDL-C.

Women had smaller LVEDD compared to men. No difference was observed in LVESD and LVEF (Table 2).

**Table 2:** Baseline laboratory and echocardiographic measurements

Variable	Men (*n* = 30)	Women (*n* = 30)	*p* value
Laboratory measurements			
Total cholesterol, mg/dl	193.37 ± 25.31	186.33 ± 21.82	0.254
Serum triglycerides, mg/dl	147.3 ± 59.78	131.4 ± 26.54	0.188
LDL-C, mg/dl	123.37 ± 20.33	118.39 ± 18.04	0.32
HDL-C, mg/dl	40.47± 5.92	41.57 ± 4.66	0.427
HbA1c, %	6.14 ± 1.63	7.43 ±2.63	0.033
Echocardiographic measurements			
LVEDD, mm	51.17 ± 4.34	48.9 ± 2.63	0.017
LVESD, mm	36.6 ± 3.78	35.3 ± 2.77	0.134
LVEF, %	53.6 ± 5.19	52.2 ± 4.26	0.258

Continuous variables are expressed as mean and standard deviation. *LDL-C* means low-density lipoprotein cholesterol, *HDL-C* means high-density lipoprotein cholesterol, *HbA1c* means glycated hemoglobin, *LVEDD* means left ventricular end-diastolic diameter, *LVESD* means left ventricular end-systolic diameter, *LVEF* means left ventricular ejection fraction

### Assessments after completion of the 12-week cardiac rehabilitation program

#### Clinical characteristics

After completion of the program, there was no difference in the number of active smokers between men and women (*p* = 0.718) (Table 3). However, the proportion of active smokers was significantly reduced from 29 (96.77%) to 5 (16.67%) for men (*p* < 0.001) and from 12 (40%) to 4 (13.33%) for women (*p* = 0.039).

**Table 3:** Clinical findings and measurements after completion of the cardiac rehabilitation program

Variable	Men (*n* = 30)	Women (*n* = 30)	*p* value
Current smoker, *n* (%)	5 (16.67%)	4 (13.33%)	0.718
BMI, kg/m^2^	28.82 ± 4.15	31.88 ± 5.22	0.015
Waist circumference, cm	99.07 ± 12.32	104.97 ± 9.29	0.041
NYHA functional class, *n* (%) Class 1 Class 2	29 (96.67%) 1 (3.33%)	29 (96.67%) 1 (3.33%)	1
Systolic BP, mmHg	114.5 ± 11.47	116.37 ± 9.87	0.502
Diastolic BP, mmHg	70.7 ± 10.03	69.37 ± 6.56	0.545
Resting heart rate, bpm	69.37 ± 8.93	73.37 ± 7.51	0.065
Total number of sessions attended, *n* (%)	19.1 ± 4.77 (79.6%)	15.7 ± 5.72 (65.4%)	0.015

Continuous variables are expressed as mean and standard deviation whereas categorical variables are expressed as number (percentage). *BMI* means body mass index, *NYHA* means New York heart association, *BP* means blood pressure

Similarly, there was no difference between men and women in the NYHA functional classes after completion of the program (*p* = 1) (Table 3). However, the proportion of patients in NYHA functional class 1 significantly increased from 19 (63.33%) to 29 (96.67%) for men (*p* = 0.002) and from 15 (50%) to 29 (96.67%) for women (*p* < 0.001).

There was a significant reduction in BMI and waist circumference for both men and women (Table 4). However, no difference was observed when comparing the magnitude of change between both groups (*p* = 0.632 for BMI, *p* = 0.599 for waist circumference). Thus, BMI and waist circumference remained higher in women at the end of the program (*p* = 0.015, *p* = 0.041 respectively) (Table 3).

**Table 4:** Paired change in clinical, laboratory and echocardiographic measurements after completion of the cardiac rehabilitation program for each gender and comparison of the magnitude of change between them

Variable	Paired change for men (*n* = 30)	Comparing paired results in men, *p* value	Paired change for women (*n* = 30)	Comparing paired results in women, *p* value	Comparing both groups for the magnitude of change, *p* value
Clinical measurements					
BMI, kg/m^2^	− 0.65 ± 0.92	< 0.001	− 0.79 ± 1.3	0.002	0.632
Waist circumference, cm	− 3 ± 3.71	< 0.001	− 3.47 ± 3.15	< 0.001	0.599
Systolic BP, mmHg	− 6.67 ± 15.22	0.023	− 12.47 ± 15.17	< 0.001	0.145
Diastolic BP, mmHg	− 5.47 ± 12.57	0.024	− 10.93 ± 8.94	< 0.001	0.058
Resting heart rate, bpm	− 3.73 ± 8.19	0.018	− 2.07 ± 12.58	0.376	0.547
Laboratory measurements					
Total cholesterol, mg/dl	− 46.87 ± 22.43	< 0.001	− 34.48 ± 16.62	< 0.001	0.018
Serum triglycerides, mg/dl	− 41.03 ± 45.62	< 0.001	− 19.03 ± 13.55	< 0.001	0.014
LDL-C, mg/dl	− 46.3 ± 21.29	< 0.001	− 37.48 ± 17.07	< 0.001	0.082
HDL-C, mg/dl	7.7 ± 4.2	< 0.001	7.45 ± 4.66	< 0.001	0.829
HbA1c, %	− 0.53 ± 1.05	0.014	− 1.33 ± 1.94	< 0.001	0.052
Echocardiographic measurements					
LVEDD, mm	0.31 ± 1.17	0.164	− 0.08 ± 1.09	0.723	0.187
LVESD, mm	− 1.03 ± 1.57	< 0.001	− 1.35± 1.41	< 0.001	0.41
LVEF, %	5.31 ± 3.01	< 0.001	5.15 ± 3.13	< 0.001	0.841

Continuous variables are expressed as mean and standard deviation. *BMI* means body mass index, *BP* means blood pressure, *LDL-C* means low-density lipoprotein cholesterol, *HDL-C* means high-density lipoprotein cholesterol, *HbA1c* means glycated hemoglobin, *LVEDD* means left ventricular end-diastolic diameter, *LVESD* means left ventricular end-systolic diameter, *LVEF* means left ventricular ejection fraction

#### Vital signs

Systolic and diastolic BP were significantly reduced for men and women, especially with women showing a highly significant reduction in diastolic BP.

No difference was observed when comparing the magnitude of change between men and women for systolic BP (*p* = 0.145). Women had more reduction in diastolic BP − 10.93 ± 8.94 vs. − 5.47 ± 12.57 mmHg although it did not quite reach statistical significance (*p* = 0.058) (Table 4).

After completion of the program, there was no difference between men and women regarding systolic and diastolic BP measurements (*p* = 0.502, *p* = 0.545 respectively) (Table 3).

The magnitude of reduction in resting heart rate was significant in men (*p* = 0.018) but not in women (*p* = 0.376). There was no difference on comparing the magnitude of change between men and women (*p* = 0.547) (Table 4). There was no difference between both groups at the end of the program regarding resting HR (*p* = 0.065) (Table 3).

#### Laboratory measurements

Serum total cholesterol, triglycerides, LDL-C, and HDL-C were significantly reduced in both groups (*p*< 0.001 for all). The magnitude of reduction in serum total cholesterol and triglycerides was more in men − 46.87 ± 22.43 vs. − 34.48 ± 16.62 mg/dl (*p* = 0.018) and − 41.03 ± 45.62 vs. − 19.03 ± 13.55 (*p* = 0.014) respectively. There was no difference in the magnitude of change between men and women regarding LDL-C and HDL-C (Table 4).

At the end of the program, there was no difference between men and women regarding serum levels of total cholesterol, triglycerides, LDL-C and HDL-C (Table 3).

There was a significant reduction in HbA1c in both men and women. Women showed more reduction in HbA1c − 1.33 ± 1.94 vs. − 0.53 ± 1.05% that was near significance (*p* = 0.052). There was no difference in HbA1c levels at the end of the program between men and women (Table 5).

**Table 5:** Laboratory and echocardiographic measurements after completion of cardiac rehabilitation program

Variable	Men (*n* = 30)	Women (*n* = 30)	*p* value
Laboratory measurements			
Total cholesterol, mg/dl	146.5 ± 12.48	151.07 ± 14.15	0.193
Serum triglycerides, mg/dl	106.27 ± 22.49	112.62 ± 19.06	0.247
LDL-C, mg/dl	77.07 ± 10.45	80.2 ± 14.56	0.345
HDL-C, mg/dl	48.17 ± 5.64	48.9 ± 4.41	0.583
HbA1c, %	5.61 ± 0.9	6.07 ± 0.98	0.074
Echocardiographic measurements			
LVEDD, mm	51.35 ± 4.01	48.96 ± 1.99	0.008
LVESD, mm	35.38 ± 4.01	34 ± 2.53	0.138
LVEF, %	59.03 ± 5.23	57.35 ± 4.53	0.209

Continuous variables are expressed as mean and standard deviation. *LDL-C* means low-density lipoprotein cholesterol, *HDL-C* means high-density lipoprotein cholesterol, *HbA1c* means glycated hemoglobin, *LVEDD* means left ventricular end-diastolic diameter, *LVESD* means left ventricular end-systolic diameter, *LVEF* means left ventricular ejection fraction

#### Echocardiographic measurements

After completion of the program, LVESD was reduced and LVEF increased for both men and women (*p* < 0.001 for all). No difference was observed for LVEDD (Table 4).

On comparing the magnitude of change between men and women, no difference was observed for LVEDD, LVESD, and LVEF (Table 4). Thus, at the end of the program LVEDD remained smaller in women 48.96 ± 1.99 vs. 51.35 ± 4.01 mm (*p* = 0.008) (Table 5).

### Adherence to the cardiac rehabilitation program

We measured adherence as the number of sessions actually attended of the total 24 sessions. Men were found to be more adherent attending 19.1 ±4 .77 (79.6%) sessions compared to women attending 15.7 ± 5.72 (65.4%) sessions (*p* = 0.015).

## Discussion

This was a prospective study conducted on 30 men and 30 women with stable ischemic heart disease who had been fully revascularized by PCI with the aim of assessing gender differences in adherence to a 12-week comprehensive cardiac rehabilitation program and differences in the expected benefits from participating in the program.

The main findings of this study are (1) men were more adherent to the cardiac rehabilitation program. (2) Women joined the program following revascularization after a significantly longer period of time (20 days on average) compared to men. (3) After completion of the program, both men and women had a significant reduction in BMI, waist circumference, blood pressure measurements, total cholesterol, triglycerides, LDL-C, HDL-C, HbA1c, and LVEDD with an increase in LVEF. Men had a significant reduction in resting HR while women did not. (4) On comparing magnitude of change after completion of the program, no differences were observed between men and women except that women had more reduction of diastolic BP and HBA1c, while men had more reduction of total cholesterol and triglyceride levels. (5) The proportion of patients in NYHA functional class 1 increased significantly after completion of the program for both men and women.

Cardiac rehabilitation currently has a class 1 recommendation in patients with stable ischemic heart disease following cardiac surgery or PCI [19–21]. The benefits of participating in the cardiac rehabilitation program provided at our institution were similar to those widely reported in the literature from other institutions [21–24].

Secondary findings of this study included differences observed in baseline characteristics of these stable, revascularized ischemic heart disease patients, where a larger proportion of women were hypertensive and diabetic, had a higher BMI, larger waist circumference, higher HbA1c, and smaller LVEDD. These differences persisted even after completion of the program, except for HbA1c level where the difference became non-significant. Smoking was less prevalent among women at baseline and a smaller proportion of women was employed. After completion of the program, there was no difference between men and women regarding the number of active smokers.

It can be concluded from these secondary findings that women have more risk factors for ischemic heart disease in general (diabetes, hypertension, obesity, and unemployment). Cardiac rehabilitation seems to significantly improve better control of diabetes as assessed by HbA1c level in women.

Several clinical studies reported that women participating in cardiac rehabilitation programs tend to have a high-risk burden, namely, hypertension, diabetes mellitus, obesity, dyslipidemia, and to a lesser extent, smoking. Studies also reported that women who complete such programs gain significant benefits [25–28].

Of note is that compared to other studies, our results show a significantly larger number of active smokers at baseline among men but not among women. This is probably a social and cultural effect as smoking for women is negatively perceived in our society and women are aware of the risks posed by smoking on their children and reproductive health which makes them refrain from smoking. Similar results were found in a study done in Iran [28], while studies performed in Western countries showed no difference between men and women in the proportion of active smokers [25–27].

In contrast to other studies, no difference was noted between men and women in the baseline lipid profile, as other studies found that women tend to have higher levels of total cholesterol, LDL-C and HDL-C [29]. The higher level of HbA1c in women at baseline was reported by others [30].

### Adherence to cardiac rehabilitation

Women in our study had a longer time to enrollment compared to men (39.17 ± 40.49 vs. 19.77 ± 10.26 days, *p* = 0.014). In a systemic review and meta-analysis examining the differences between men and women regarding enrollment in a cardiac rehabilitation program that included 297,219 participants from the year 2000 to 2011, it was concluded that women are 36% less likely to be enrolled in a program and thus less likely to achieve the morbidity and mortality benefits of cardiac rehabilitation. The enrollment rate for men was 45 ± 18.5% vs. 38.5 ± 20.7% for women (*p* < 0.001) [30].

Using the number of sessions attended as an indicator of adherence to the program, we found that women were less adherent as they attended 15.7 ± 5.72 (65.4%) sessions compared to 19.10 ± 4.77 (79.6%) sessions for men (*p* = 0.015). Local cultural, social, and educational barriers that face women may be responsible for this outcome. A meta-analysis examining the differences between men and women regarding adherence to a cardiac rehabilitation program that included 8176 participants from fourteen studies reported that the mean adherence rate was 68.6% for men and 64.2% for women. It was concluded that, in general, patients adhere to over two thirds of sessions. However, adherence is significantly lower in women (*p* < 0.001) [31].

Another study examining the reasons for withdrawal from a 12-month cardiac rehabilitation program in Canada examined 1089 women and 4833 men. Researchers reported that women were more likely than men to withdraw from the program. Causes were attributed to medical problems, especially musculoskeletal problems. Family obligations and transportation were important barriers for women, while work and lack of interest were barriers for men [32].

The same message can be concluded from a recent systemic review examining the barriers and possible solutions to the limited participation of women in cardiac rehabilitation programs. Researchers stated that a complex combination of modifiable and non-modifiable social, economic, medical, psychological, demographic, and medical challenges face women and recommended the search for solutions such as providing home-based programs designed for women to work on this gender gap [33].

### Changes observed after completion of the program

Several studies reported changes (benefits) after completion of the program similar to ours. Gender disparity was investigated in a study on 12,976 patients of which 69% completed a cardiac rehabilitation program. They reported that both men and women greatly improved but women were less likely to reach target goals of the American Heart Association/American College of Cardiology (AHA/ACC) in serum triglyceride levels and HbA1c, while they were more likely to achieve them for HDL-C. No gender differences were observed regarding achieving AHA/ACC goals of BP, total cholesterol, LDL-C, BMI, smoking cessation, and medication use [34].

Another study on 858 patients assessed the prevalence of women in cardiac rehabilitation programs and their response to the program. They reported that women represented 24% of participants in their program and that improvement was observed in total cholesterol, triglycerides, LDL-C, HDL-C, fasting blood sugar, HbA1c, and N-terminal pro-brain natriuretic peptide levels in the blood, in addition to, improvements in functional capacity and heart rate recovery [29].

### Study limitations

The limitations of the current study are that it comes from a single medical center with a relatively small number of patients. We did not measure the quality of life parameters and did not assess depressive symptoms which have been shown to affect outcome and adherence to cardiac rehabilitation programs in other studies. While nutritional and lifestyle advice was provided to all, actual adherence to such measures at home could not be controlled. The results of this study should be considered with the confounding effect of possible changes in the medication regimen during the 3 months period taken into account.

## Conclusion

Men are more adherent to cardiac rehabilitation programs. The recruitment of women is significantly delayed. Women have a higher cardiovascular risk burden in the form of the prevalence of diabetes, hypertension, and obesity. Completion of a cardiac rehabilitation program causes a reduction in BMI, waist circumference, blood pressure measurements, total cholesterol, triglycerides, LDL-C, HDL-C, HbA1c, and LVEDD with an increase in LVEF in both genders. Men show more reduction in resting HR, total cholesterol, and triglyceride levels while women show more reduction in diastolic BP and HBA1c.

## Data Availability

The datasets used and analyzed during the current study are available from the corresponding author on reasonable request

## Abbreviations

BMI: Body mass index
HbA1c: Glycated hemoglobin
LDL-C: Low-density lipoprotein cholesterol
HDL-C: High-density lipoprotein cholesterol
LV: Left ventricle
EF: Ejection fraction
LVEDD: LV end-diastolic diameter
LVESD: LV end-systolic diameter
HR: Heart rate
BP: Blood pressure
PCI: Percutaneous coronary intervention
NYHA: New York Heart Association
SPSS: Statistical package for social science
AHA/ACC: American Heart Association/American College of Cardiology
